# Acoustic coordinated reset therapy for tinnitus with perceptually relevant frequency spacing and levels

**DOI:** 10.1038/s41598-019-49945-w

**Published:** 2019-09-20

**Authors:** Peter A. Tass, Alexander N. Silchenko, Gerald R. Popelka

**Affiliations:** 1 Department of Neurosurgery, Stanford University, Stanford, CA USA; 2 Institute of Neuroscience and Medicine – Neuromodulation (INM-7), Jülich Research Center, Jülich, Germany; 3 Department of Otolaryngology-Head and Neck Surgery, Stanford University, Stanford, CA USA

**Keywords:** Neurological disorders, Computational neuroscience

## Abstract

Acoustic coordinated reset (CR) therapy based on neuromodulation and neuroplasticity principles has been proposed for the treatment of tonal tinnitus. The original therapy involved periodic delivery of randomly ordered sequences of four low-level tones centered around the frequency of a tone that matched the tinnitus pitch, f_T_, with fixed ratios relative to f_T_ and delivered several hours/day over several weeks. Here we transform the original CR tone selection method to a more perceptually-relevant equivalent rectangular bandwidth (ERB) frequency scale, the ERB_N_-number scale. Specifically, we provide a mathematical model that enables calculation of CR tones that accounts for f_T_- and hearing loss-related ERB widening and ERB overlaps and gaps of CR tone alignments. Further, the model ensures symmetric CR tone alignments based on modelling studies that indicate the effect is optimal if the CR stimuli are symmetrically spaced relative to the tinnitus-related population of abnormally synchronized cortical neurons to activate the adjacent sub-populations. We also present experimentally testable ERB-based CR tone alignment strategies and explain how to use the ERB-based model in experiments, clinical studies, other types of tinnitus sound treatment such as tailor-made notch music training and limitations of our approach.

## Introduction

Tinnitus is the perception of sound in the absence of a sound source external to the person^1,2^. Some forms of tinnitus, called “objective” or “secondary” tinnitus, can arise from an identifiable sound source in the body, for example arterial blood flow. However, most cases of tinnitus cannot be explained in terms of a physical sound source. “Subjective” or “primary” tinnitus is used to describe the perception of sound that does not have any obvious acoustic origin. Subjective tinnitus is often associated with damage to the peripheral auditory system, but it probably reflects more central nervous system changes that follow the peripheral damage, specifically deafferentiation^3–5^. One such change is abnormal neural synchrony; neurons that normally would show relatively independent patterns of firing may have abnormal responses that are highly synchronized^6,7^. A second possible change is tonotopic reorganization^4^; the remapping of acoustic frequency to neural position within an array of tonotopically organized neurons may be altered. However, there is no clear evidence for tonotopic map changes in the auditory cortex of humans with tinnitus^8^ or partial hearing loss^9^, although such reorganization may occur for severe or profound hearing loss of long duration, or when there are dead regions in the cochlea^10,11^.

The tinnitus percept is highly variable including self-reported differences in temporal characteristics (constant vs intermittent), perceptual locations (monaural, binaural and central), loudness (low to high) and subjective descriptors (humming, buzzing, ringing, banging, clicking, sea-like, machine-like, and squeaking and many other subjective descriptors)^12^. Regardless of these large ranges, the percept can be objectively and reliably characterized by careful psychoacoustic studies of pitch matching, loudness matching and masking using frequency-specific and broad band calibrated external stimuli. The most comprehensive and largest data set describing these characteristics^12^ reported a frequency-specific tinnitus in over 96.5% of cases with the tinnitus pitch matched to a tone (<1000 Hz, 27.6%; between 1001 and 3000 Hz, 10.3%; between 3001 and 6000 Hz, 17.3%; between 6001 and 8000 Hz, 10.3%; and >8000 Hz, 31%) and the small remainder requiring a non-frequency specific signal (white noise, 3.5%). Though it is possible for pitch matching to be affected by multiple pitch components of the percept, the primary pitch is likely the dominant factor associated with tonotopic locations in the auditory pathways.

The concept of coordinated reset (CR) neuromodulation was developed based on computational studies^13^. It aims at a desynchronization-induced synaptic modulation by delivery of specific spatio-temporal stimulus patterns, ultimately inducing sustained, long-lasting desynchronization^14^ via neuroplasticity principles and applies to several neurologic conditions. According to computational studies, the CR approach can be applied by means of both invasive and non-invasive stimulation^15,16^. Invasive electrical CR deep brain stimulation induced long-lasting motor effects in Parkinsonian monkeys^17,18^ and Parkinson’s patients^19^. Analogously, non-invasive acoustic CR stimulation induced long-lasting tinnitus loudness and annoyance reduction effects^20^ based on the concept that tonal tinnitus arises primarily from abnormal neural synchrony across a defined array of tonotopically organized neurons^16^.

The theoretical basis of CR therapy for tinnitus is to desynchronize the abnormal neural activity via the sequential presentation of sinusoidal tones with frequencies on either side of the frequency of a tone that is matched in pitch to the tinnitus^16,20^. A key aim of this approach is to reduce or eliminate the tinnitus percept for a long period after the therapy ceases. A proof of concept study of acoustic CR therapy in 63 patients with chronic tonal tinnitus showed that 12 weeks of acoustic CR treatment, delivered 4–6 h/day, caused significant therapeutic effects compared to baseline. The therapeutic effects persisted through a preplanned 4-week therapy pause and showed sustained long-term effects after 10 months of therapy, giving rise to 75% responders^20^. Therapeutic effects included a significant reduction of tinnitus questionnaire (TQ) scores^21^, and Visual Analog Scale (VAS) scores for loudness (VAS-L) and annoyance (VAS-A) compared to initial scores. In addition, acoustic CR therapy showed significant reductions in the frequency of the tinnitus percept and in tinnitus-related abnormal synchrony and effective neural connectivity as assessed by high density EEG recordings^20,22,23^. The clinical effects of acoustic CR treatment achieved in the prospective, randomized, single blind, placebo-controlled proof of concept study by Tass *et al*.^20^ were reproduced in two open label studies without sham control group^24,25^.

None of the computational studies published so far predicted optimal values of the overlaps of the spatial stimulation profiles (see Discussion). In particular, none of these existing neural network models have taken into account spatial stimulation profiles required to model basic tuning characteristics of the auditory cortex such as co-tuned excitation and inhibition as opposed to lateral inhibition^26^. By the same token, hearing loss-related spatially varying stimulation profiles at the CR tones have not been taken into account in computational studies. However, in all 1D, 2D and 3D neural network models used for the development of CR stimulation, *the stimulation profiles of the different stimulation sites were symmetrical*, i.e. overlaps of neighboring stimulation sites were identical, no matter whether the different spatial stimulation profiles were actually overlapping or not (see Discussion). Accordingly, we hypothesize that CR tones that enable symmetric stimulation profile overlaps and gaps of the stimulated cortical sub-populations of neurons will be favorable for desynchronization.

Filters play a key role in fundamental concepts of audiology and psychoacoustics^27^. For instance, the concept of critical bands introduced an “auditory filter” created by the cochlea in the inner ear^27^. A second tone delivered within the band of audio frequencies belonging to an auditory filter interferes with the perception of a first tone due to auditory masking^27^. The equivalent rectangular bandwidth (ERB) is another concept associated with the auditory filter^28,29^. The ERB uses a rectangular band-pass filter approximation, where the ERB passes the same amount of energy as its corresponding auditory filter, and provides the relationship between the auditory filter, frequency, and the critical bandwidth^28,29^.

The purpose of the current report is to develop a more advanced, auditory filter-based theoretical basis for the selection of the acoustic CR therapy tones that incorporates both a more perceptually relevant frequency scale than fixed ratios relative to the frequency matched to the tinnitus pitch, as well as known changes to the perceptually relevant frequency scale associated with stimulus level and sensorineural hearing loss. Based on this theoretical approach we analyze the selection of CR tones used so far and design novel CR therapy tone arrangements that can be tested experimentally.

## Results

### ERB framework

The hypothesis aims to select CR tones that stimulate different brain sites e.g. within the primary auditory cortex with symmetrically overlapping stimulation profiles. Because the spatial extent of the area with abnormal neuronal synchrony is unknown, in a first step, the CR tones are symmetrically placed around the frequency of the tinnitus, with two CR tones above and two below the tinnitus frequency^20^. The tonotopic organization that originates in the cochlea is preserved at all levels of the auditory nervous system up to the primary auditory cortex^30^. Hence, the location of the sub-population of cortical neurons excited by a given CR tone will depend on the frequency of the CR tone. The spatial extent of the sub-population of neurons will depend mainly on the frequency selectivity of the cochlea at the place tuned to the frequency of the tone. This selectivity can be quantified using the bandwidth of the auditory filter centered at that frequency^29^. This bandwidth increases with increasing sound level and with increasing sensorineural hearing loss at that frequency^31–33^. For optimal tinnitus reduction, we hypothesize that the relative overlap of the spatial stimulation profiles of neighboring CR tones be identical and symmetrically arranged relative to the profile of the frequency of a tone matched to the pitch of the tinnitus, designated here as f_T_^13,16,34^.

The frequencies of the four tones used in a proof of concept study^20^ and two open label studies^24,25^ were specified as fixed percentages relative to f_T_, with two placed below f_T_ and two placed above f_T_. The frequencies of the tones were defined by:

1 $${{\rm{f}}}_{1}=0.766{{\rm{f}}}_{{\rm{T}}}$$

2 $${{\rm{f}}}_{2}=0.9{{\rm{f}}}_{{\rm{T}}}$$

3 $${{\rm{f}}}_{3}=1.1{{\rm{f}}}_{{\rm{T}}}$$

4 $${{\rm{f}}}_{4}=1.4{{\rm{f}}}_{{\rm{T}}}$$

However, it is not clear whether the selected frequencies were optimally spaced. The selection of frequencies based on a perceptually relevant frequency scale might be more appropriate. In the next section we consider the effect of selecting the frequencies of the tones based on such a scale.

We use a frequency scale based on the equivalent rectangular bandwidth (ERB) of the auditory filter, as determined from masking experiments using notched noise or spectrally rippled noise with human listeners^28,29^. The average value of the ERB for young listeners with normal audiometric thresholds measured at moderate sound levels is denoted ERB_N_^1^. Its value in Hz is given by:

5 $${{\rm{ERB}}}_{{\rm{N}}}=24.7(0.00437\,{\rm{f}}+1)$$

where *f* is the center frequency in Hz^29^. This equation gives a good prediction of ERB values estimated psychoacoustically using masking experiments for center frequencies spanning almost the entire range of human hearing from about 50 Hz^35,36^ to 15,000 Hz^37^.

### Estimation of ERB widths from audiometric thresholds

The tonotopic mapping in the cochlea is preserved at higher levels in the auditory system^30^, so constant steps on the ERB scale correspond to approximately constant spatial distances for neural sub-populations that are tonotopically organized, including the auditory cortex. Recent data suggest that tonotopic spatial maps do not change in the auditory cortex of humans with tinnitus^8^ or with partial sensorineural hearing loss^9^. In other words, the mapping of frequency to position in the auditory cortex is not markedly changed by changes in hearing sensitivity associated with sensorineural hearing loss. However, there is considerable evidence showing that the ERB widths of the auditory filters increase with increasing signal level and therefore with increasing sensorineural hearing loss. For a given degree of sensorineural hearing loss at a specific frequency there can be marked individual variations in the value of the ERB, but, on average, the ERB increases with increasing sensorineural hearing loss^32,33,38^. The spatial extent of the tonotopically organized neural sub-population in the auditory cortex activated by a tone of a given frequency can reasonably be assumed to be proportional to the ERB of the auditory filter centered at that frequency. On average, this ERB will increase with increasing sensorineural hearing loss at that frequency, hence increasing the spatial spread of the activated neural sub-population.

We estimated the ERB-width values at low sensation levels (SLs) for any frequency based on the hearing level at that frequency, using data presented by Moore *et al*.^32^. Moore *et al*.^32^ provided data on the value of the ERB for center frequencies of 2000, 4000, and 6000 Hz and audiometric thresholds between 0 and 80 dB HL. Based on their data, the dependence of the ERB on the hearing loss *h* for the range between 0 and 50 dB HL can be modelled with good accuracy by:

6 $${\rm{ERB}}({h})={{\rm{ERB}}}_{{\rm{N}}}\ast \{1+[{h}/(50{\rm{dB}}\,{\rm{HL}})]\}$$

For hearing loss greater 50 dB HL there was no such clear relationship^32^. For frequencies below 2000 and above 6000 Hz, the relationship between amount of hearing loss and auditory filter bandwidth is less well established and this approximation may be less accurate. However, more than 60% of cases of tonal tinnitus have values of f_T_ that fall within the range 2000–6000 Hz^12^ and there is no reason to expect that the approximation will be substantially different for f_T_ outside of this frequency range.

**Figure 1:**
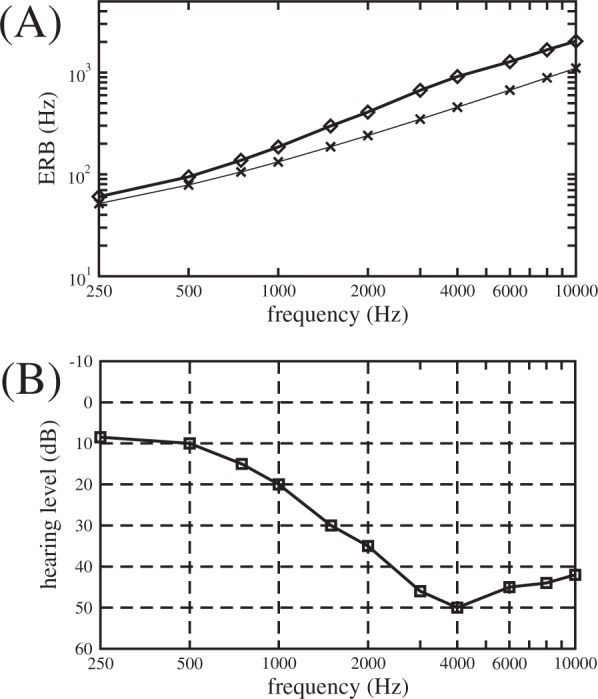
ERBs and corresponding hearing thresholds. The lower panel (**B**) shows a typical^24^ example audiogram in dB HL (linearly interpolated on a logarithmic frequency axis). Symbols indicate frequencies at which the hearing threshold was measured. The upper panel (**A**) shows the values of ERB_N_ for normal hearing (thin line with crosses) and the values of the ERB estimated from the audiometric thresholds in the lower plot (thicker line with diamonds).

**Figure 2:**
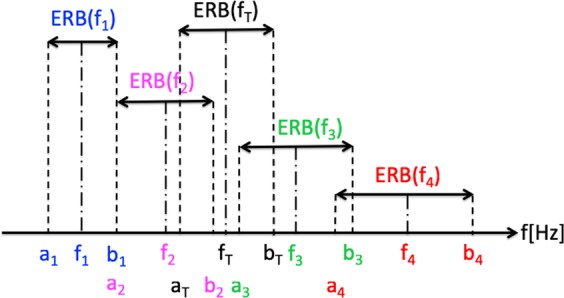
Alignment of CR tones, tinnitus tone and corresponding ERBs. The symbols used to designate the band edges and center frequencies of the ERB-wide bands around the center frequencies of each of the four CR tones and f_T_.

The effect of hearing threshold on ERB widths is illustrated in Fig. 1. The upper panel (Fig. 1A) shows the values of ERB_N_ for normal hearing according to Eq. 5 (thin line with crosses) and for ERB (*h*) according to Eq. 6 (thicker line with diamonds) for a representative audiogram for a typical tinnitus patient (Fig. 1B) as a function of frequency. Based on Eq. 6, overlaps or gaps between ERBs of neighboring CR tones, ERB (f_j_) and ERB (f_k_), as well as between ERBs of CR tones ERB (f_j_) and the tinnitus ERB (f_T_) can be analyzed and quantified (Fig. 2, see Methods).

### Standard CR stimulation

**Figure 3:**
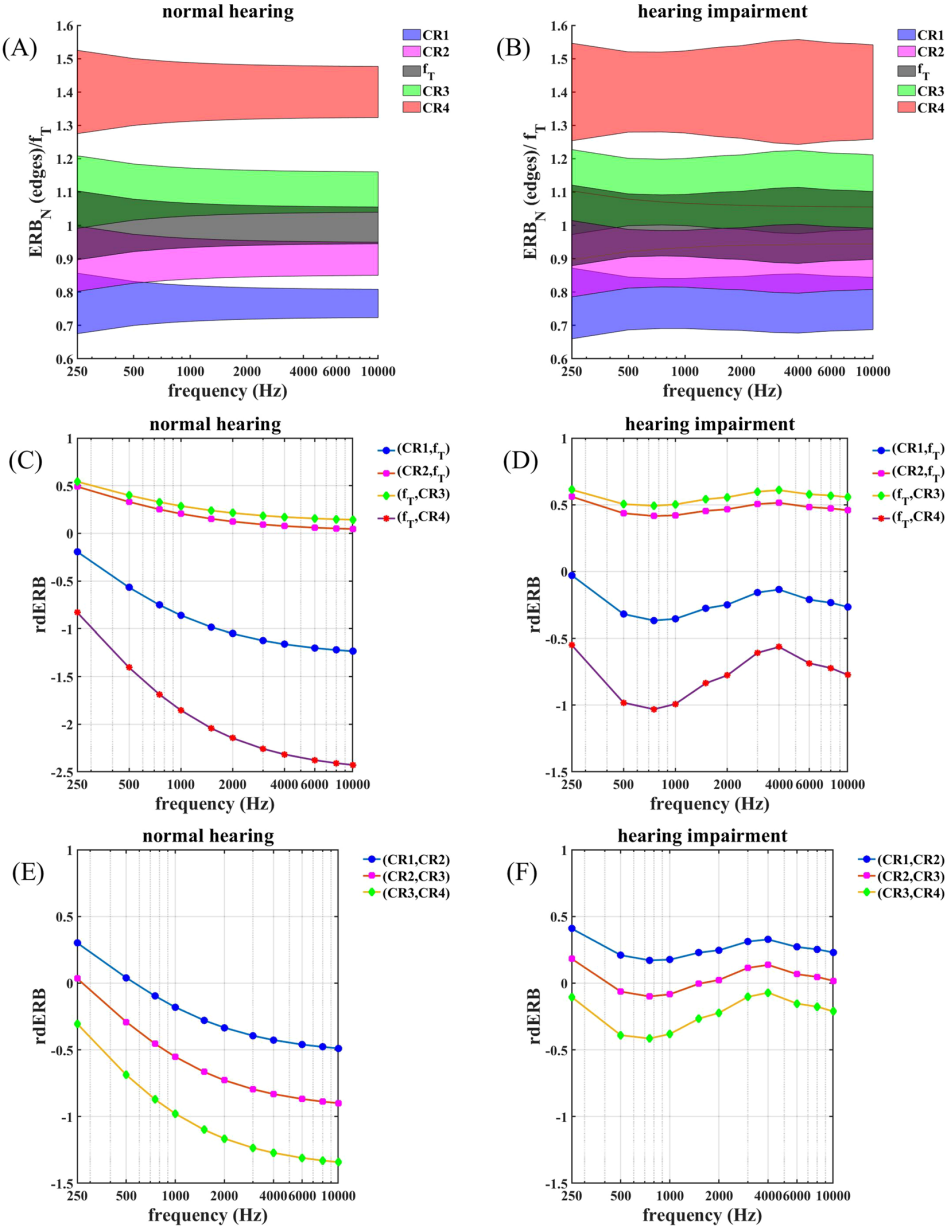
Alignment of CR therapy ERBs and tinnitus ERB: Illustration of the effect of f_T_ and of sensorineural hearing loss on the alignment of the ERB-wide bands centered around the frequencies of each of the four CR tones and the band centered around f_T_. The left panels (**A**,**C**,**E**) are for the case of normal hearing and the right panels (**B**,**D**,**F**) are for sensorineural hearing loss shown in the audiogram in Fig. 1B. (**A**,**B**) The abscissa shows the value of f_T_, and the ordinate shows the frequencies of the band edges relative to f_T_. The shaded areas show the extent of the ERB-wide band around the center frequency of each CR tone and around f_T_. (**C**,**D**) The abscissa shows the value of f_T_, and the ordinate shows the relative overlaps of the ERB-wide band around CR tone 1, …, 4 and the ERB-wide band around f_T_ as defined by Eqs 11–14. (E,F) Panels show the relative overlap of ERB-wide bands of neighboring CR tones as introduced by Eqs 15–17 against f_T_. Positive values of relative overlaps correspond to overlapping ERBs, whereas negative values indicate gaps between ERBs, and zero means that the ERB edges coincide.

Figure 3 illustrates the effect of f_T_ and of hearing loss on the alignment and overlap of the ERB-wide bands centered around the frequencies of each of the CR tones and f_T_, as a function of f_T_. Figure 3A,C,E are for a case of normal hearing with *h* = 0 dB HL for all audiometric frequencies, and Fig. 3B,D,F are for a case of sensorineural hearing loss using the audiogram shown in Fig. 1B where the magnitude of the hearing loss changes with audiometric frequency. In Fig. 3A,B the abscissa shows frequency in Hz and the ordinate shows the frequencies of the ERB band edges relative to the ERB band edges of f_T_. The colored areas show the extent of the ERB-wide band around the frequency of each CR tone and around f_T_. Let us first consider Fig. 3A. Based on the ERB_N_ scale, the mutual arrangement of the standard CR therapy tones, given by Eqs 1–4 ^20^, as well as their relation to the tinnitus ERB, is asymmetrical spacing.

#### Alignment of CR tones relative to the tinnitus ERB at f_T_ (normal hearing)

The ERB-wide bands around f_2_ and f_3_ overlap only slightly with the ERB-wide band around f_T_ for high values of f_T_, because ERB_N_ is relatively small (about 11% of the center frequency) at high frequencies (Fig. 3A). However, for low frequencies, the value of ERB_N_ relative to the center frequency increases (to about 20% of the center frequency at 250 Hz), so the overlap of the ERB-wide bands around f_2_ and f_3_ with the ERB-wide band around f_T_ increases (Fig. 3A). The alignment of CR tones 2 and 3 relative to the tinnitus tone is asymmetrical, and this asymmetry depends on f_T_ in that the relative overlap rdERB (3,f_T_) exceeds rdERB (2,f_T_) for all values of f_T_, and the overlap ratio rdERB (3,f_T_)/rdERB (2,f_T_) increases with increasing f_T_ (Fig. 3C). Other than with CR tones 2 and 3, the ERB-wide bands around f_1_ and f_4_ do not overlap with the ERB-wide band around f_T_. The alignment of CR tones 1 and 4 relative to the tinnitus tone is asymmetrical in that rdERB (f_T_, 4) = (b_T_ − a_4_)/(b_T_ − a_T_), the gap between the ERB-wide band around f_4_ and the ERB-wide band around f_T_, exceeds rdERB (1, f_T_) = (b_1_ − a_T_)/(b_T_ − a_T_), the gap between the ERB-wide band around f_1_ and the ERB-wide band around f_T_, by a factor of at least 2 (Fig. 3C). Furthermore, this gap decreases with increasing f_T_, so that the asymmetry of the alignment of CR tones 1 and 4 relative to f_T_ depends on f_T_.

#### Alignment of CR tones relative to each other (normal hearing)

The mutual alignment of the CR tones is asymmetrical, and the asymmetry depends on f_T_ (Fig. 3A,E). The ERB-wide band around f_1_ and f_2_ overlaps up to 560 Hz (Fig. 3A,E). For greater values of f_T_ there is a gap between both ERBs. The ERB-wide bands of CR tones 2 and 3 have a minor overlap up to 270 Hz and a gap in between for greater values of f_T_ (Fig. 3A,E). The ERB-wide bands of CR tones 3 and 4 do not overlap for all values of f_T_ (Fig. 3A,E). The gap between CR tones 3 and 4 increases with increasing f_T_ (Fig. 3A,E). Because only neighboring CR tones can overlap it is sufficient to calculate the relative overlap of neighboring ERBs as defined by Eqs 15–17 and 18–20.

To study the impact of hearing loss on the alignment of the ERB-wide bands of the CR tones and the ERB-wide band of the tone matched to the pitch of the tinnitus frequency, we consider a case of hearing loss using the audiogram shown in Fig. 1B.

#### Alignment of CR tones relative to the tinnitus ERB at f_T_ (hearing loss)

The ERBs of the CR tones as well as the tinnitus ERB are broadened at frequencies f_T_ that have hearing loss. Accordingly, the overlap of the ERB-wide bands around f_2_ and f_3_ with the ERB-wide band around f_T_ is greater than in the case of normal hearing and ranges between 40% and 60%, depending on f_T_ (Fig. 3B,D). Consequently, the tinnitus ERB is covered by the ERB-wide bands around f_2_ and f_3_ except for f_T_ between 450 Hz and 1500 Hz. The gap between the ERB of CR tone 1 and the tinnitus ERB is smaller, especially for values of f_T_ with greater hearing loss (Fig. 3B,D). The gap between the ERB of tone 1 and the tinnitus ERB is smaller than in the case of normal hearing, in particular at 4000 Hz where the hearing loss is the greatest (Fig. 3B,D).

#### Alignment of CR tones relative to each other (hearing loss)

The hearing loss-induced ERB-broadening causes a tighter alignment of the ERBs of the CR tones. The ERB-wide bands of f_1_ and f_2_ overlap for all values of f_T_. The ERBs of CR tones 2 and 3 overlap at lower values of f_T_ as well as for values of f_T_ with greater hearing loss (Fig. 3B,D). The gap between the ERBs of CR tones 3 and 4 is markedly reduced compared to the normal hearing case, and both ERB bands nearly coincide at 4000 Hz, corresponding to most hearing loss (Fig. 3B,D).

In summary, due to the hearing loss given by the audiogram in Fig. 1B, the ERBs of CR tones 1 and 2 as well as the ERBs of CR tones 2 and 3 overlap for all values of f_T_. The tinnitus ERB is covered by the ERBs of CR tones 2 and 3 over a wide range of values of f_T_. Unlike in the normal hearing case, only the ERB of CR tone 4 does not overlap with the ERB of its neighboring CR tone. The mutual overlaps and gaps depend on the amount of hearing loss and, hence, on f_T_.

### Design of hearing threshold adapted CR tone alignment

In principle, with the methods from Section 3 a variety of different types of CR tone alignments can be designed. To constrain possible options, based on previous studies (see Section 2) we hypothesize that CR tone alignments with identical relative overlap between adjacent CR tone ERBs may be favorable for desynchronization. The procedure for the calculation of the CR tones requires the following input:

- The audiogram in dB HL (including standardized frequencies greater than the typical 8000 Hz limit in most audiograms). We perform a linear interpolation on a logarithmic frequency axis.
- The tinnitus frequency f_T_.
- The actual value of rdERB, the relative ERB overlap, as defined by Eqs 15–17 or 18–20, where rdERB (1,2) = rdERB (2,3) = rdERB (3,4).
- The number of CR tones. Standard CR therapy employs 4 tones^20^. However, translating findings from computational studies in the field of deep brain stimulation (DBS)^39^, different numbers of tones might be optimal and should, in particular, be adapted to the spatial dimension of the synchronized neuronal population in relation to the ERB-related area of neural tissue activated at the different simulation sites.

### Tinnitus-centered CR tone alignment

**Figure 4:**
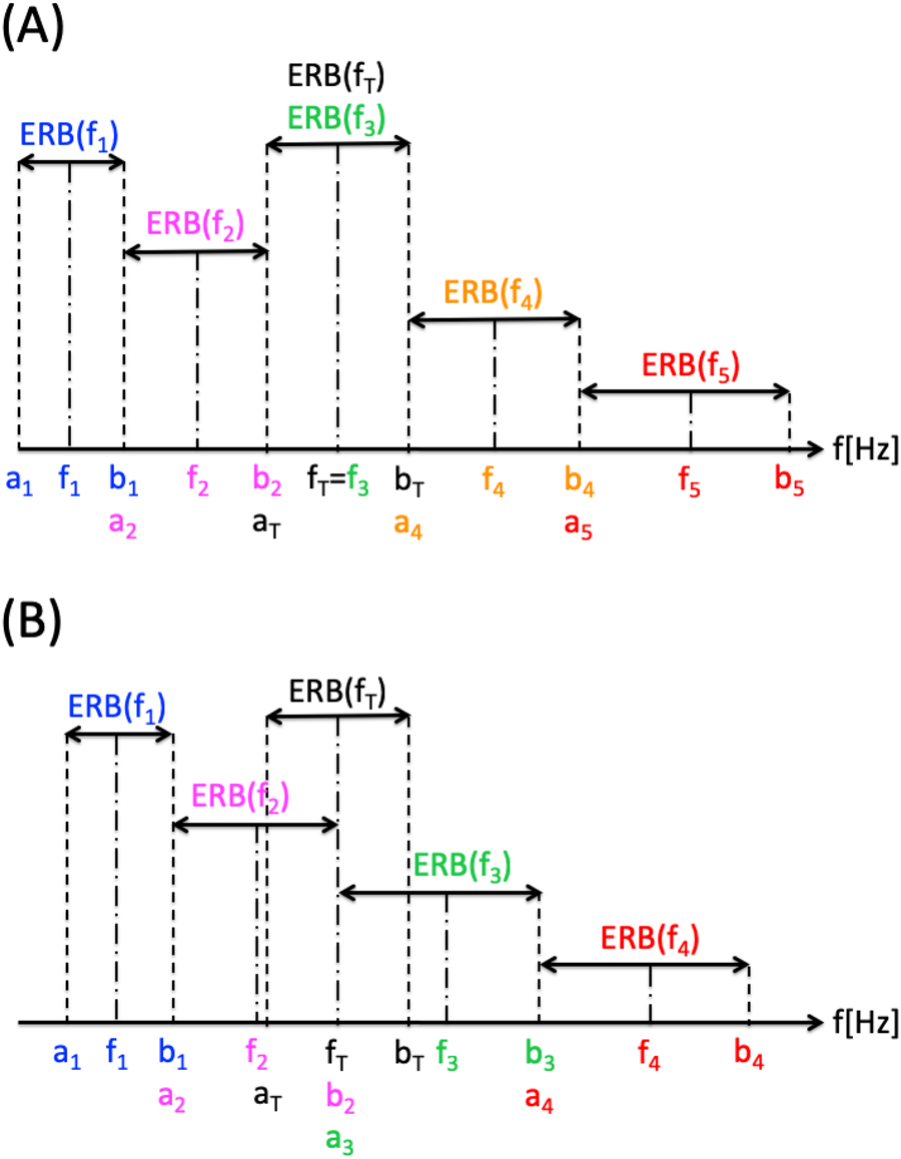
Different types of CR tone alignments with coincident ERB edges. (**A**) Tinnitus-centered alignment of 5 CR tones, where the center CR tone equals the tinnitus tone, f_3_ = f_T_. (**B**) Tinnitus-enclosing alignment of 4 CR tones, where the coinciding ERB edges of CR tones 2 and 3 are identical with the tinnitus tone f_T_.

In contrast to the tone alignment of standard acoustic CR stimulation, for this type of alignment one CR tone equals the tinnitus tone f_T_. In the case of a symmetric alignment of e.g. five CR tones, the center CR tone (f_3_) equals the tinnitus tone f_T_, as illustrated in Fig. 4A for coinciding ERB edges (i.e. a_j+1_ = b_j_ and hence rdERB (j, j + 1) = 0). The two lower tones (f_1_, f_2_) and the two higher tones (f_4_, f_5_) are grouped around f_T_ such that their ERB band edges coincide (Fig. 4A).

### Tinnitus-centered CR tone alignment with coinciding ERB bands

**Figure 5:**
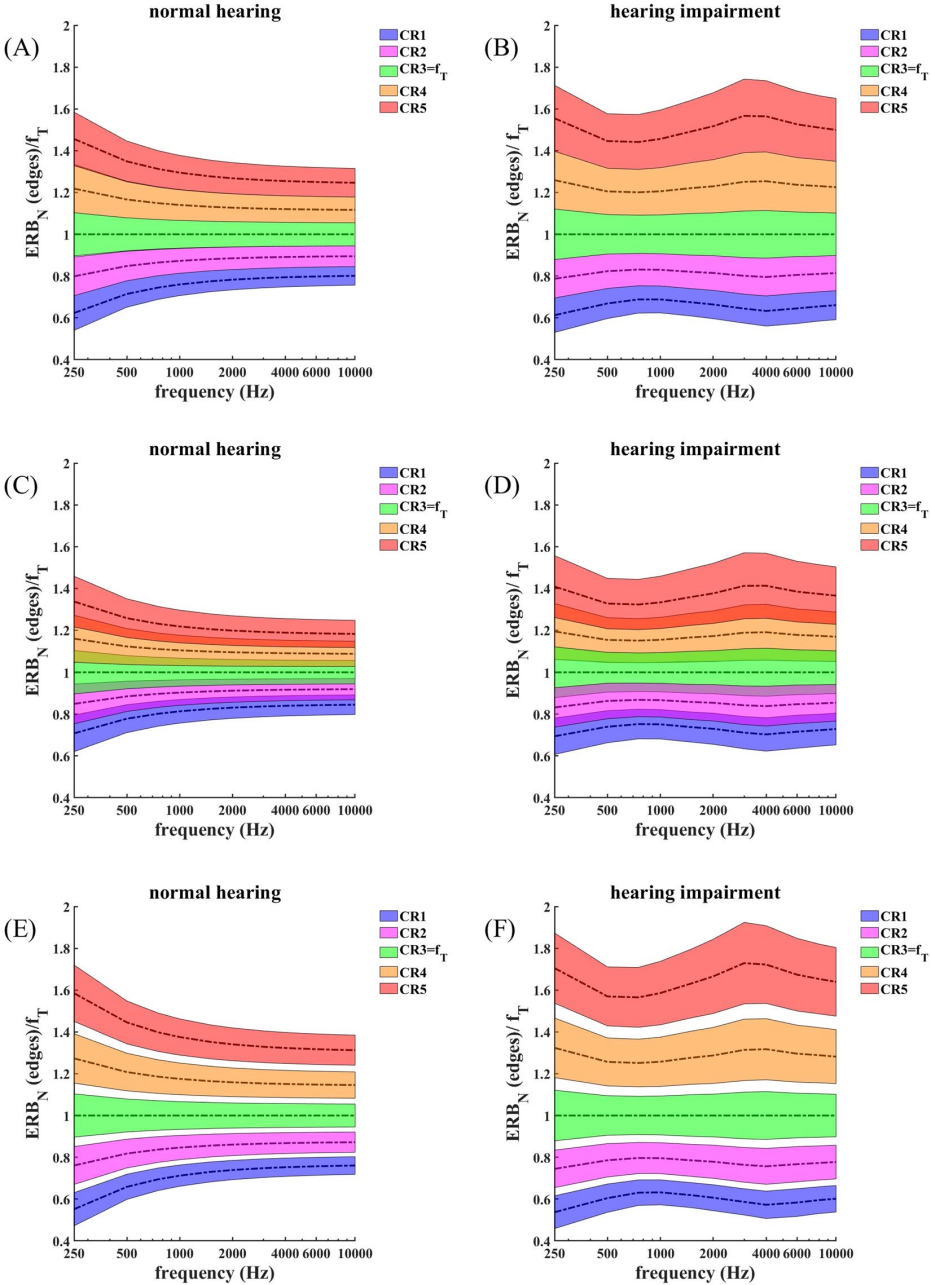
Different types of tinnitus-centered CR tone alignments. (**A**–**F**) The ordinate shows the frequencies of the ERB band edges relative to f_T_. The abscissa shows the value of f_T_. The shaded areas illustrate the ERB-wide bands around the CR tones and around f_T_. The corresponding CR tones relative to f_T_, i.e. f_1_/f_T_, f_2_/f_T_, f_3_/f_T_, f_4_/f_T_, f_5_/f_T_ are illustrated by dot and dash lines. The center CR tone equals the tinnitus tone: f_3_ = f_T_. The same format as in Fig. 3A,B. (**A**,**C**,**E**) refer to normal hearing. (**B**,**D**,**F**) refer to sensorineural hearing loss given by the audiogram in Fig. 1B. (**A**,**B**) ERB bands of neighboring CR tones coincide. (**C**,**D**) ERB bands of neighboring CR tones overlap by 25% relative to neighboring CR tone ERB bands as defined by Eqs 15–17. (**E**,**F**) ERB bands of neighboring CR tones are separated by a 25% gap relative to neighboring CR tone ERB bands as defined by Eqs 15–17.

For normal hearing (Fig. 5A) as well as the hearing loss shown in Fig. 1B (Fig. 5B) we vary the tinnitus frequency f_T_ between 250 Hz and 10,000 Hz and determine the CR tones f_1_, …, f_5_ with coinciding neighboring ERB edges. Only for normal hearing the ERB width decreases, and the frequency ratios f_j_/f_T_ tend to 1 from above (f_4_, f_5_) and below (f_1_, f_2_) as f_T_ increases (Fig. 5A). In contrast, this pattern no longer holds in the hearing loss case, where the ERB width attains maxima at 3000 Hz and 4000 Hz for CR tones 2, 3, 4, and 5 (Fig. 5B).

### Tinnitus-centered CR tone alignment with ERB overlaps

For normal hearing (Fig. 5C) as well as for the same audiogram from Fig. 1B (Fig. 5D) and tinnitus frequency f_T_ between 250 Hz and 10,000 Hz we now calculate the CR tones f_1_, …, f_5_ with neighboring ERBs symmetically overlapping by 25%, i.e. rdERB (1,2) = rdERB (2,3) = rdERB (3,4) = 0.25, with rdERB (j, k) from Eqs 15–17. Due to the 25% ERB overlap the CR frequency ratios f_j_/f_T_ are, in general, closer to 1 (for j = 1, 2, 4, 5). The overall picture with narrowing ERB width is preserved for the normal hearing case (Fig. 5C). By the same token, in the hearing loss case maximal ERB widths as well as maximal frequency ratios f_j_/f_T_ (for j = 4, 5) and minimal frequency ratios f_j_/f_T_ (for j = 1, 2) are observed at 3000 Hz and 4000 Hz (Fig. 5D).

### Tinnitus-centered CR tone alignment with ERB gaps

To illustrate identical relative ERB gaps, we consider the normal hearing case (Fig. 5E) and use the audiogram from Fig. 1B (Fig. 5F), vary the tinnitus frequency f_T_ between 250 Hz and 10,000 Hz and determine the CR tones f_1_, …, f_5_ such that the gaps between neighboring ERBs are identical and equal to 25%, i.e. rdERB (1, 2) = rdERB (2, 3) = rdERB (3, 4) = −0.25, with rdERB (j, k) from Eqs 15–17. As a consequence of the 25% ERB gap the CR frequency ratios f_j_/f_T_ are, in general, more spread, (for j = 1, 2, 4, 5). However, for the normal hearing case ERB widths narrow with increasing tinnitus frequency f_T_ (Fig. 5E). As before, in the hearing loss case maximal ERB widths together with maximal frequency ratios f_j_/f_T_ (for j = 4, 5) and minimal frequency ratios f_j_/f_T_ (for j = 1, 2) are observed at 3000 Hz and 4000 Hz (Fig. 5F).

### Tinnitus-enclosing CR tone alignment

**Figure 6:**
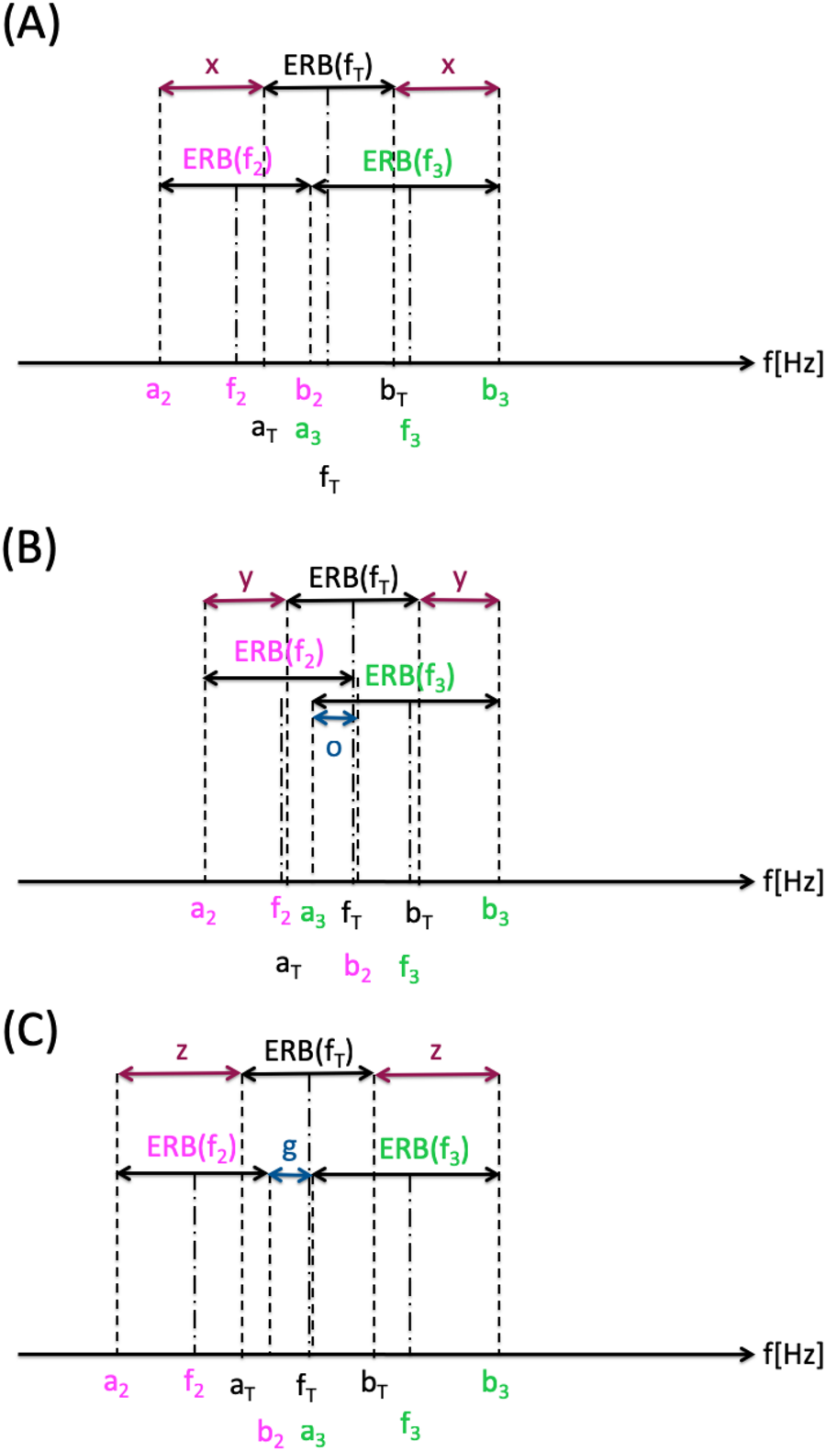
Different types of tinnitus-enclosing ERB-balanced alignments of CR tones 2 and 3 around the tinnitus ERB. (**A**) Coinciding ERB edges of CR tones 2 and 3. (**B**) ERBs of CR tones 2 and 3 display an overlap (‘o’, blue double arrow) of width 25% relative to the ERB of CR tone 2 (calculated by Eq. 16). (**C**) ERBs of CR tones 2 and 3 separated by gap (‘g’, blue double arrow) of width 25% relative to the ERB of CR tone 2 (calculated by Eq. 16). The tinnitus ERB is located in the middle of the entire interval formed by the ERBs of CR tones 2 and 3, so that f_T_ is in the middle, and f_T_ = (a_2_ + b_3_)/2 holds (**A**–**C**), no matter whether ERB band edges coincide (**A**), ERBs overlap (**B**) or ERBs are separated by a gap (**C**). Consequently, the tinnitus ERB is symmetrically aligned with identical space on both sides (x in A, y in B, z in C).

Alternatively, CR tones also can be symmetrically aligned on both sides of the tinnitus tone f_T_, where none of the CR tones coincides with f_T_. For instance, we may align the 4 CR tones relative to f_T_ so that the coinciding ERB edges of CR tones 2 and 3 coincide with the ERB edges of the tinnitus tone f_T_, i.e. a_3_ = b_2_ = f_T_ (Fig. 4B). Alternatively, we can design an ERB-balanced alignment of CR tones 2 and 3 relative to the tinnitus tone f_T_ that works whether the ERBs of CR tones 2 and 3 overlap, share an edge or are separated by a gap. To this end, we require the tinnitus ERB to be located in the middle of the entire interval formed by the ERBs of CR tones 2 and 3, so that f_T_ is in the middle, which means that f_T_ = (a_2_ + b_3_)/2 has to be fulfilled. This condition holds irrespective of whether ERB band edges coincide (Fig. 6A), overlap (Fig. 6B) or are separated by a gap (Fig. 6C).

### Tinnitus-enclosing CR tone alignment with coinciding ERB edges and f_T_ coinciding with ERB edges

**Figure 7:**
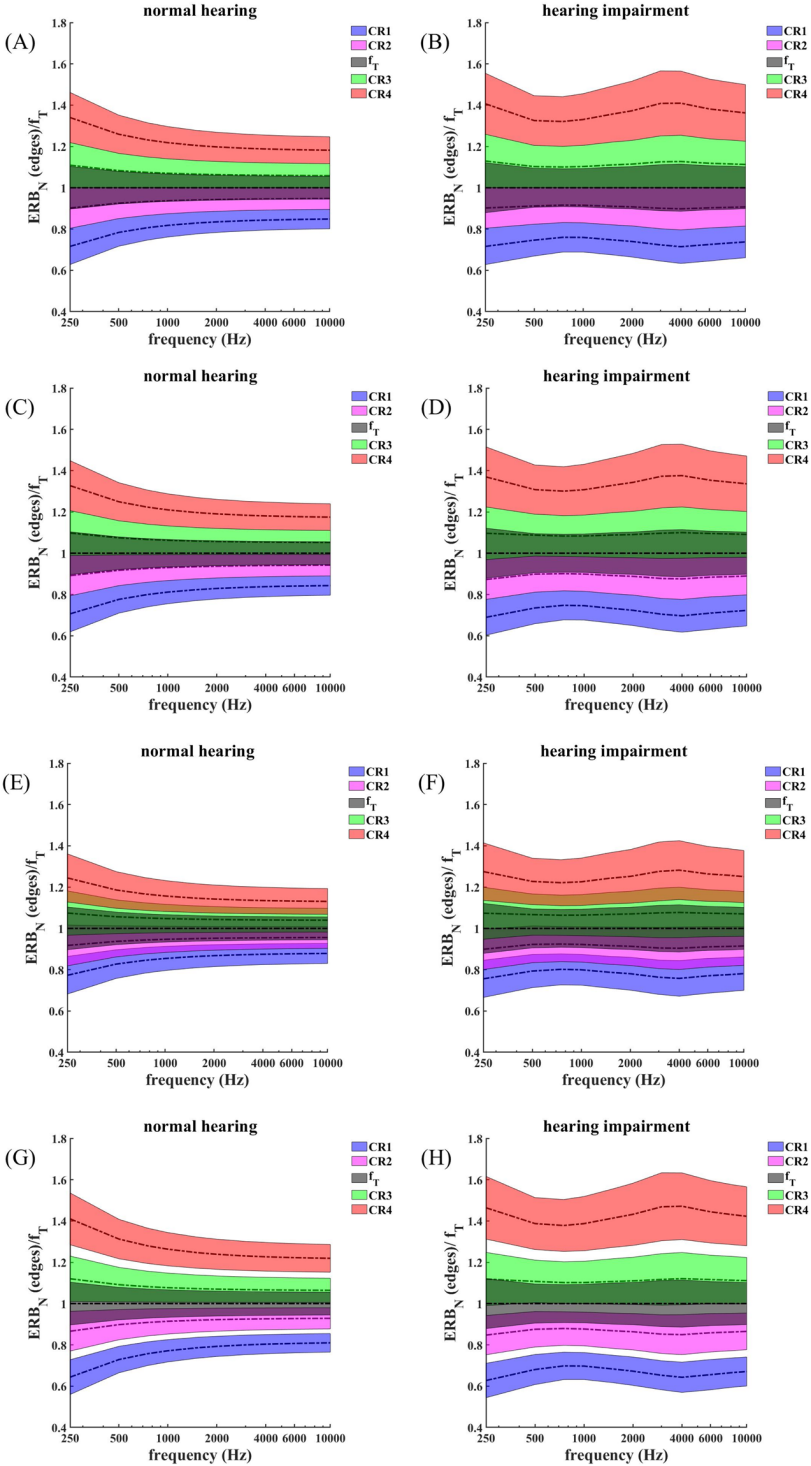
Different types of tinnitus-enclosing CR tone alignments. A-H: The frequencies of the ERB band edges relative to f_T_ are plotted against f_T_. Shading illustrates ERB-wide bands. The corresponding CR tones f_1_, f_2_, f_3_, f_4_ and the tinnitus frequency f_T_ are illustrated by dot and dash lines. Same format as in Fig. 5. Two CR tones are placed on either side of the tinnitus frequency f_T_. (**A**,**C**,**E**,**G**) refer to normal hearing. (**B**,**D**,**F**,**H**) refer to sensorineural hearing loss given by the audiogram in Fig. 1B. (**A**–**D**) ERB bands of neighboring CR tones coincide, where the ERB edges of CR tones 2 and 3 coincide with f_T_ (**A**,**B**), or the ERB balancing condition f_T_ = (a_2_ + b_3_)/2 is fulfilled for each f_T_ (**C**,**D**). (**E**,**F**) Relative overlap of neighboring CR tone ERB bands is 25%. G,H: Neighboring CR tone ERB bands are separated by a relative gap of 25%. (**C**–**H**) The ERB balancing condition f_T_ = (a_2_ + b_3_)/2 is fulfilled for each f_T_.

For the case of normal hearing (Fig. 7A) and for the audiogram shown in Fig. 1B (Fig. 7B) and tinnitus frequency f_T_ between 250 Hz and 10,000 Hz we determine the CR tones f_1_, …, f_4_ so that the ERB edges of CR tones 2 and 3 coincide with the ERB edges of the tinnitus tone f_T_ (Fig. 4B). In addition, ERB edges of neighboring CR tones also coincide (Fig. 4B). For normal hearing the ERB width decreases and the frequency ratios f_j_/fT continuously tend to 1 from above (f_3_, f_4_) and below (f_1_, f_2_) as f_T_ increases (Fig. 7A). For the hearing loss case this pattern no longer holds, and the ERB width attains maxima, e.g., at 3000 Hz and 4000 Hz for CR tones 1–4 (Fig. 7B).

### Tinnitus-enclosing CR tone alignment with coinciding ERB edges and with ERB-based balancing around f_T_

To illustrate the effect of an ERB width-related symmetrical spacing of the CR tones 2 and 3 in relation to the tinnitus ERB (Fig. 6A), for normal hearing (Fig. 7C) and for the audiogram from Fig. 1B (Fig. 7D) and tinnitus frequency f_T_ between 250 Hz and 10,000 Hz, we determine the CR tones f_1_, …, f_4_ so that the ERB symmetrical spacing condition f_T_ = (a_2_ + b_3_)/2 is fulfilled for each f_T_ (Fig. 6A). Due to the ERB symmetrical spacing around f_T_, the latter does not coincide with the ERB edges of CR tones 2 and 3, and the entire CR tone and ERB alignment is slightly shifted towards smaller frequency ratios (Fig. 7C,D). Apart from that shift, the overall ERB and CR tone alignment is very similar to the case without ERB symmetrical spacing around f_T_ (compare Fig. 7A,B with 7 C,D).

### Tinnitus-enclosing CR tone alignment with ERB overlaps and with ERB-based symmetry around f_T_

Here we design CR tones with overlapping ERBs for normal hearing (Fig. 7E) and for the audiogram from Fig. 1B (Fig. 7F) and tinnitus frequency f_T_ between 250 Hz and 10,000 Hz. We determine the CR tones f_1_, …, f_4_ so that all relative overlaps of neighboring ERBs are 25%, i.e. rdERB (1,2) = rdERB (2,3) = rdERB (3,4) = 0.25, with rdERB (j,k) from Eqs 15–17 (Fig. 7E,F). For the placement of the CR tones relative to the tinnitus frequency f_T_ we use the ERB symmetry condition f_T_ = (a_2_ + b_3_)/2 for each f_T_ (Fig. 6B). Except for the ERB overlaps, the overall pattern of CR tone and ERB arrangement (Fig. 7E,F) is similar to the case without gaps (Fig. 7C,D).

### Tinnitus-enclosing CR tone alignment with ERB gaps and with ERB-based symmetry around f_T_

In contrast to coinciding (Fig. 7C,D) or overlapping (Fig. 7E,F) CR-tone ERBs, here we calculate CR tones f_1_, …, f_4_ with symmetric ERB gaps for normal hearing (Fig. 7G) and for the hearing loss case from Fig. 1B (Fig. 7H) and tinnitus frequency f_T_ between 250 Hz and 10,000 Hz. To this end, relative gaps between neighboring CR tone ERBs are chosen to be identical at 25%, i.e. rdERB (1,2) = rdERB (2,3) = rdERB (3,4) = −0.25, where rdERB (j,k) is given by Eqs 15–17 (Fig. 7G,H). As in Fig. 7C–F we employ the ERB symmetry condition f_T_ = (a_2_ + b_3_)/2 for each f_T_ (Fig. 6C). Similarly as in Fig. 7C–F the ERB balancing around f_T_ slightly shifts the entire CR tone and ERB arrangement towards smaller frequency ratios. Apart from the gaps the overall CR tone and ERB arrangement (Fig. 7G,H) resembles that with coinciding (Fig. 7C,D) or overlapping (Fig. 7E,F) ERB edges.

## Discussion

Tass *et al*.^20^ proposed an acoustic CR neuromodulation therapy for tinnitus based on neuroscience principles and computational modelling^13,14,16^. The computational studies published so far do not provide predictions about optimal values of the overlaps of the spatial stimulation profiles in spatial brain coordinates (see below). However, previous studies indicate that favorable desynchronizing effects can be obtained with spatially symmetrically arranged stimulation profiles, where two cases were considered: (i) The overlap of the stimulation profiles of all pairs of neighboring stimulation sites in the brain was either identical, or (ii) the stimulation profiles of all pairs of neighboring stimulation sites were non-overlapping and equidistant (see below). Accordingly, we hypothesized that because of the strict tonotopic organization of the auditory system from the sensory end organ to the auditory cortex, favorable alignments of the CR tones on the frequency axis should enable an identical overlap of neighboring spatial stimulation profiles in brain spatial coordinates.

To this end, we propose here a more perceptually relevant transformation of the frequency scale for determination of optimal frequency spacing of the CR tones, the ERB_N_-number scale^29^. We assumed that the mapping of frequency to position in the cortical tonotopic map is not affected by mild to moderate sensorineural hearing loss, but that the widths of the auditory filters are affected by sensorineural hearing loss, and that these widths influence the degree of overlap of the ERB band around the tinnitus frequency f_T_ and the ERB bands around each of the CR tones, denoted dERB number (j, f_T_), where j denotes the number of the CR tone. We also considered the change in ERB bandwidth associated with the frequency dependent individual hearing levels of the patient. The theoretical model proposed here advances the original theory^16,20^ by incorporating a more relevant perceptual frequency scale, the ERB, as well as changes in ERB values associated with stimulus level and magnitude of sensorineural hearing loss up to 50 dB HL. With this ERB-based approach we analyzed the alignment of the original four CR tones, specified as fixed percentages relative to f_T_ (f_1_ = 0.766f_T_, f_2_ = 0.9f_T_, f_3_ = 1.1f_T_, f_4_ = 1.4f_T_), employed in a proof of concept study^20^ and two subsequent studies^24,25^.

We used the hearing threshold adapted ERB(*h*) as defined in Eq. 6 instead of a hearing-threshold adapted ERB number scale because for frequency dependent hearing threshold *h*(f), in general, there is no closed-form formula for ERB number (f). Furthermore, we are focusing on ERB relationships in the vicinity of the tinnitus frequency and, hence, do not need to count the number of ERBs subjacent to f_T_.

The mutual alignment of the CR tones as well their alignment relative to the tinnitus ERB are highly asymmetric with respect to mutual distance, overlap with tinnitus ERB, and overlap of neighboring CR tones, in the case of both normal hearing and hearing loss (see Section 4.1). In particular, irrespective of the hearing loss, the ERB overlaps of neighboring CR tones are typically not identical (Fig. 3E,F). Furthermore, the alignment pattern of tinnitus ERB and CR tone ERBs strongly vary with tinnitus frequency f_T_ (Fig. 3C,D). Because the majority of patients undergo a significant reduction of f_T_ in the course of a 12-week acoustic CR therapy^20,24,25^, the ERB overlaps will vary in the course of the treatment, too. If the efficacy of the CR therapy depends on the ERB alignment of the tones and, especially, on the ERB overlaps, treatment efficacy will vary during the course of the treatment, too. The pronounced and, in addition, time-varying asymmetry of the CR tone alignment might be a reason why effects of the original acoustic CR therapy needed weeks to build up^20,24,25^ and may even explain variability of treatment effects across patients.

Other factors to be considered for enhancing therapeutic effects of acoustic CR neuromodulation include overall therapeutic stimulation equivalent to therapeutic “dose”. Longer stimulation per day that could be implemented into hearing aids being worn by the patient up to 18 hours/day rather than use of a custom device that requires removal of an existing hearing aid already worn by many tinnitus patients that itself provides some tinnitus benefit^2^. Use of a modified hearing aid to provide the tinnitus therapy tones could also objectively record the total therapy duration using “data logging”, a common feature of contemporary hearing aids. Development of algorithms implemented by contemporary hearing aids also can optimize CR tone selection that include improvements in automated pitch matching and automated incorporation of hearing levels. By the same token, a clinically established dosage regimen, e.g. using ERB-based principles of CR tones alignment might further increase the efficacy of acoustic CR stimulation as illustrated in a computational study^40^. However, all of these potential improvements still require solid clinical testing.

Because we do not know the exact spatial dimension and location of tinnitus-related abnormally synchronized populations in the individual patient’s brain and their exact relationship to the tinnitus ERB, the optimal number of CR tones as well the most appropriate alignment strategy, sparing the tinnitus frequency f_T_ (Fig. 7) as opposed to including a CR tone at f_T_ (Fig. 5), remain to be determined. In fact, the number of CR tones as well as their alignment relative to f_T_ might have to be personalized and recalibrated during the course of the treatment to achieve optimal stimulation effects. Establishing such methods requires controlled comparative clinical studies, where either different patient groups are treated with different ERB-based CR tone alignments and/or number of CR tones or where one or more patient groups receive different ERB-based CR tone alignments and/or number of CR tones in a cross-over protocol. To reduce related efforts, duration and costs, it might be favorable to first select optimal candidate parameters by means of acute studies with EEG recordings, i.e. by studying electrophysiological effects and after-effects of short stimulation epochs of, say 15 min as, for instance, in a study comparing two different types of acoustic CR stimulation protocols and a sham (i.e. detuned CR) stimulation protocol^41^. Electrophysiological after-effects, in particular, the post-stimulus time required for resynchronization of e.g. delta band oscillations in the auditory cortex, turned out to distinguish between effective and less effective versions of acoustic CR stimulation^41^. The most promising parameters revealed in such an acute study should ultimately be tested in a proof of concept study, e.g. providing a comparison to standard acoustic CR stimulation.

Standard acoustic CR stimulation with adequately pitch matched tinnitus tone causes acute clinical effects after 15 min^20,41^. Clinical improvement can readily and quickly be assessed by means of VAS scores for tinnitus loudness and tinnitus annoyance^20,41^. An additional, technical more sophisticated means for calibration for acoustic CR stimulation is to measure sound stimulation-induced reduction of EEG delta band power (over the auditory cortex or of reconstructed auditory cortex currents underlying EEG) and prolonged delta resynchronization^41^. These EEG effects evolve on the same time scale as the clinical effects, i.e. 15 min^20,41^. In clinical studies it still remains to be shown whether the EEG-based calibration is clinically superior to the simple VAS-based calibration, in particular given that an EEG-based calibration is more time consuming. However, already the simple VAS-based calibration could provide an adequate means to compare different stimulation parameter settings. For instance, by means of a therapeutic sound stimulation app the patient might test different settings and select the favorable one which provides strongest tinnitus relief. For example, one could start with an approach that is reasonably close to the standard acoustic CR^20^ and aims at improving the treatment outcome step-by-step by comparisons: One could start with 4 CR tones with ERB-based tinnitus-enclosing alignment with coinciding ERB bands of neighboring CR tones, sparing the tinnitus frequency f_T_ (Fig. 7C,D). After 15 min sound stimulation the patient turns stimulation off for 10 min. After the 10 min pause the patient resumes sound stimulation for 15 min, where only one parameter is changed, for instance, by setting the relative ERB overlap to 25% (Fig. 7E,F). During the 15 min stimulation epochs as well as during the 10 min pauses tinnitus loudness and tinnitus annoyance are evaluated by VAS scores (as it was done in an EEG study^41^). Based on clinically relevant sound stimulation-induced VAS reduction^42^ the more efficient setting will be chosen, say, the coinciding ERB bands of neighboring CR tones might be better. VAS-based pairwise selections of stimulation settings differing by just one parameter value, e.g. the relative ERB overlap, may be repeatedly be performed and ultimately provide psychophysically optimized stimulation parameters. This approach can equally be applied to optimize other stimulation parameters, for instance, the number of CR tones (e.g. by comparing VAS reduction achieved with 4 as opposed to 6 CR tones) or different tone alignment schemes.

To put our manuscript and subsequent, secondary efforts (see below) into perspective, we note that hearing threshold adapted CR stimulation was invented at Stanford by GRP and PAT^43^. Their patent application^43^ comprises the model for hearing loss-induced ERB widening (Eq. 6), ERB overlap and gap calculations (Eqs 11–20) using ERB edges (Eqs 7–10) and an ERB overlap-based CR tone alignment strategy^43^. Only one publication has been reported that did not use the original CR tones, a study in 25 patients where CR tone frequencies and levels were adapted to hearing thesholds^44^. According to their Fig. 1 in this study^44^ the ratios between the CR tone frequencies and the tinnitus frequency f_j_/f_T_ were (i) adapted to stimulation intensities of up to 80 dB HL, (ii) did not depend on the tinnitus frequency, and (iii) were based on the assumption that tuning curves of CR tones should optimally overlap by 25–30%^44^. The exact procedure used by^44^ was not fully disclosed. However, our model for the hearing threshold-related ERB widening (Eq. 6) presented here (i) is valid only up to hearing loss of 50 dB HL^32^ (see below), (ii) is based on the concept that the ratios between the CR tone frequencies and the tinnitus frequency f_j_/f_T_ significantly depend on the tinnitus frequency, because the ERB width depends on both tinnitus frequency f_T_ and hearing loss (Eqs 5 and 6)^32^ and (iii) notes that neither computational nor pre-clinical data suggest that an overlap of the tuning curves of CR tones of 25–30% could be considered as optimal (see below). The differences between our hypothesis presented here and what was used in this clinical study^44^ means that a meaningful comparison is not possible. In addition, the lack of results obtained with standard CR stimulation^20^ make it difficult to infer any superiority of the results reported in this clinical study^44^.

There are *limitations* to our approach. Eq. 6 is key, because it describes the dependence of the ERB on the hearing loss *h* for the range between 0 and 50 dB HL. For hearing loss exceeding 50 dB HL existing experimental data do not support this model^32^. Accordingly, the approach presented here should only be applied for cases up to 50 dB HL. By the same token, Eq. 6 is based on experimental data on the value of the ERB for center frequencies of 2000, 4000, and 6000 Hz^32^. For frequencies outside the range 2000–6000 Hz, the relationship between amount of hearing loss and auditory filter bandwidth still has to be established. Accordingly, for tinnitus frequencies that are outside the range 2000–6000 Hz Eq. 6 might not be valid and, hence, our approach has to be used with great care. However, because more than 60% of cases of tonal tinnitus fall within the range 2000–6000 Hz^12^, our approach is justified for a relevant portion of tonal tinnitus patients.

Based on our ERB analysis, the arrangement of the original CR tones used for standard acoustic CR neuromodulation^20^ is highly asymmetric. Let us consider this aspect from a computational point of view. CR stimulation was computationally developed by means of a number of computational studies in qualitatively different 1D model neural networks^34,39,45,46^, 1D neural networks with periodic boundary conditions (i.e. rings)^15,16^, 2D^13,14,47–51^ and 3D neural networks^52,53^. In these computational-based studies equally spaced linear^39,45,53^, circular^15,16,54,55^ or rectangular^13,14,40,47–51^ arrangements of the stimulation sites were used. The sub-populations, stimulated by the different stimulation sites, either did not overlap at all^13,45^, or their overlap depended on stimulation amplitude, spatial arrangement of stimulation sites and spatial stimulation profile^13–16,39,40,46–58^. To this end, different stimulation profiles were used to model current spread in brain tissue caused by electrical stimulation^59,60^.

The stimulation amplitude turned out to be an important stimulation parameter. For instance, in accordance with theoretical predictions^34^, invasive electrical CR deep brain stimulation at weak intensities (i.e. with pulse amplitudes of only a third of that used for standard deep brain stimulation) delivered to the subthalamic nucleus in Parkinsonian monkeys caused a long-lasting relief of motor symptoms, persisting for several weeks after cessation of stimulation^17,18^. In contrast, the after-effect of electrical CR deep brain stimulation at high intensities (i.e. with pulse amplitudes identical to those used for standard deep brain stimulation) was limited to only the first five days after cessation of stimulation^17^. Stimulation amplitude matters for CR stimulation, irrespective of the stimulation modality. In general, at higher stimulation amplitudes the dynamics of the single stimulated neuron may qualitatively change, e.g. firing rates may change significantly, and model neurons may be blocked^34,56^. In addition, the stimulation amplitude affects the spatial overlap of the different stimulated sub-populations. In a neural network model without spike-timing-dependent plasticity (STDP), the stimulation amplitude and the spatial decay rate of the stimulus profile were varied, while the equidistantly positioned stimulation sites were fixed^34^. The spatial decay rate was an approximation of an electric field of a line charge of finite length, relevant for electrical stimulation of brain tissue^60^. For fixed stimulation amplitude, larger spatial overlaps of the stimulation profiles caused a more pronounced desynchronization (in terms of a uniform distribution of the neurons’ phases), whereas smaller spatial overlaps of the stimulation profiles led to cluster states (neurons do not fire coincidentally, but are arranged in different sub-populations firing at equally distributed times)^34^. For fixed stimulation profiles, the actual spatial overlap of the sub-populations stimulated at different stimulation sites increases with an increase of the stimulation amplitude^34^. Hence, for a wide range of the overlap of the stimulation profiles as well as the stimulation intensity and, hence, the actual overlap of the stimulated sub-populations, CR stimulation effectively caused a desynchronization^34^. However, the presence of spike timing dependent plasticity (STDP) may fundamentally change the spontaneous (i.e. stimulation-free) as well as stimulation-induced dynamics of neural networks^56^. For instance, in the presence of STDP, CR stimulation may be effective without causing phase resets of sub-populations^56^. So far, in neural networks with STDP the impact of the spatial decay rate of the stimulus profile on the desynchronizing impact of CR stimulation has not been investigated systematically. Also, in^34^ CR stimulation with fixed CR sequences was used, whereas acoustic CR stimulation employs CR sequences randomly varying from one cycle to another^20^. CR variants that differ with respect to their sequence variation have different stimulation effects^56^.

In summary, symmetric stimulation site arrangements have been used to study and optimize CR stimulation. However, stimulation site arrangements with pronounced asymmetries might, in principle, be feasible, but have hardly been studied computationally so far. Hence, computational studies into that direction might generate relevant predictions that can be tested experimentally.

The acoustic CR therapy requires a frequency-specific tinnitus with one or a few dominant tones. In a study in 1440 patients a frequency-specific tinnitus was reported in over 96.5% of cases^12^. In the presence of several tones of similar loudness and/or annoyance, the highest tone could serve as first target tinnitus tone f_T_, since in the majority of patients the tinnitus tone decreases in the course of standard acoustic CR therapy^20,25^. However, a clinical study is required to investigate whether acoustic CR with ERB-based tone spacing may also cause pronounced changes of tinnitus pitch.

Our new approach allows well defined studies to be conducted to assess the effectiveness of stimulus settings. It is expected that optimization of stimulus protocols using the proposed modifications that consider a perceptually relevant frequency scale and individual hearing thresholds will improve the effectiveness of the original CR therapy by reducing the amount of therapy needed either per day, or number of weeks, and possibly increasing the permanency of the intervention.

Our approach may also be applied to other sound treatments. For instance, tailor-made notch music training (TMNMT) aims to reduce tinnitus-related brain activity by means of lateral inhibition by activating neurons in the neighborhood of the tinnitus frequency f_T_, but sparing activation of neurons at f_T_^61^. To this end, patients listened to notch-filtered music where a frequency band of 1/2-octave around the individual tinnitus frequency f_T_ was removed from the power spectrum of the music, whereas the frequencies at the edge of the notch were amplified^61^. In a double-blind randomized controlled trial of 3 months duration in 100 patients suffering from chronic tonal tinnitus TMNMT was compared with a placebo (moving frequency notch) condition^62^. No effect was revealed for the primary outcome measures^62^. The width of the notch filter and the width of the amplified edge enclosing the notch were neither adapted to the tinnitus frequency f_T_ nor to the hearing threshold. Hence, one might hypothesize that the efficacy of TMNMT may increase by adapting its filter characteristics to tinnitus frequency f_T_ as well as hearing threshold.

## Methods

### Estimation of ERB overlap

The degree of overlap between the ERB-wide frequency band centered on the frequency of a given CR tone and the ERB-wide frequency band centered at f_T_ is determined by two factors: the separation of the ERB edges of the two bands and the ERB widths of the two bands. For a normal-hearing ear and for sounds presented at a moderate level, for example, an ERB band centered at 934 Hz would have band edges at 872 Hz and 997 Hz, while an ERB band centered at 1066 Hz would have band edges at 996 Hz and 1136 Hz. However, for an ear with sensorineural hearing loss, the width of each band is increased, while the mapping of the center frequency of the ERB remains approximately the same. Hence the ERB-wide bands around the two frequencies will overlap by an amount depending on the two frequencies and the magnitude of the hearing loss.

We next consider the notation illustrated in Fig. 2 that shows a representative f_T_, (black font) and the four CR tones (blue, pink, green and red font) determined by Eqs 1–4 on a linear frequency scale. Centered on each of these frequencies is the ERB bandwidth using Eq. 5 for each tone with lines extended to the frequency axis that define the center frequency and the frequencies at the edges of the bands. The symbols a_j_ and b_j_ denote the lower and upper edge frequencies of the ERB for the j-th CR tone (j = 1–4) at center frequency f_j_. The symbols a_T_ and b_T_ denote the lower and upper edge frequencies of the ERB-wide band centered at f_T_. The frequencies at the upper and lower edges of the ERB band centered around CR tone j, a_j_ and b_j_, were calculated as:

7 $${{\rm{a}}}_{{\rm{j}}}={{\rm{f}}}_{{\rm{j}}}-0.5\ast {\rm{ERB}}({\rm{h}}\,{\rm{at}}\,{{\rm{f}}}_{{\rm{j}}})$$

8 $${{\rm{b}}}_{{\rm{j}}}={{\rm{f}}}_{{\rm{j}}}+0.5\ast {\rm{ERB}}({\rm{h}}\,{\rm{at}}\,{{\rm{f}}}_{{\rm{j}}})$$

Similarly, the frequencies at the upper and lower edges of the ERB band centered around f_T_, a_T_ and b_T_, were calculated as:

9 $${{\rm{a}}}_{{\rm{T}}}={{\rm{f}}}_{{\rm{T}}}-0.5\ast {\rm{ERB}}({\rm{h}}\,{\rm{at}}\,{{\rm{f}}}_{{\rm{T}}})$$

10 $${{\rm{b}}}_{{\rm{T}}}={{\rm{f}}}_{{\rm{T}}}+0.5\ast {\rm{ERB}}({\rm{h}}\,{\rm{at}}\,{{\rm{f}}}_{{\rm{T}}})$$

We calculate the degree of overlap of the ERB band centered at the frequency of a given CR tone (f_1_, f_2_, f_3_, and f_4_) with the ERB band centered at f_T_. For example, the overlap of the upper edge of the ERB band centered at f_2_ with the ERB band centered at f_T_ is b_2_ − a_T_. If this quantity is negative, then there is a gap and no overlap. If this quantity is zero, the band edges coincide. Positive values indicate the amount of overlap.

Because ERB_N_ increases with frequency *f* according to Eq. 5, already in the normal hearing condition there is a fundamental asymmetry of the spatial “stimulation profiles” and, hence, the corresponding overlaps have not yet been taken into account in computational studies (see Sec. 2). Other ERB arrangements and corresponding ERB overlaps are conceivable and can be used for computational and experimental studies.

### Tinnitus-centered ERB overlap calculation

Considering the tinnitus ERB to be the target of the CR intervention, we calculate the relative overlap of the ERB-wide band around CR tone j and the ERB-wide band around f_T_ by

11 $${\rm{rdERB}}\,(1,\,{{\rm{f}}}_{{\rm{T}}})=({{\rm{b}}}_{1}-{{\rm{a}}}_{{\rm{T}}})/({{\rm{b}}}_{{\rm{T}}}-{{\rm{a}}}_{{\rm{T}}})$$

12 $${\rm{rdERB}}\,(2,\,{{\rm{f}}}_{{\rm{T}}})=({{\rm{b}}}_{2}-{{\rm{a}}}_{{\rm{T}}})/({{\rm{b}}}_{{\rm{T}}}-{{\rm{a}}}_{{\rm{T}}})$$

13 $${\rm{rdERB}}\,({{\rm{f}}}_{{\rm{T}}},\,3)=({{\rm{b}}}_{{\rm{T}}}-{{\rm{a}}}_{3})/({{\rm{b}}}_{{\rm{T}}}-{{\rm{a}}}_{{\rm{T}}})$$

14 $${\rm{rdERB}}\,({{\rm{f}}}_{{\rm{T}}},\,4)=({{\rm{b}}}_{{\rm{T}}}-{{\rm{a}}}_{4})/({{\rm{b}}}_{{\rm{T}}}-{{\rm{a}}}_{{\rm{T}}}).$$

### Therapy tone-centered ERB overlap calculation

Aiming at symmetric ERB overlaps of neighboring CR tones, we determine the relative overlap of the ERB-wide band around CR tone j and the ERB-wide band around tone k by

15 $${\rm{rdERB}}\,(1,\,2)=({{\rm{b}}}_{1}-{{\rm{a}}}_{2})/\,{\rm{\min }}[({{\rm{b}}}_{1}-{{\rm{a}}}_{1}),\,({{\rm{b}}}_{2}-{{\rm{a}}}_{2})]$$

16 $${\rm{rdERB}}\,(2,\,3)=({{\rm{b}}}_{2}-{{\rm{a}}}_{3})/\,{\rm{\min }}[({{\rm{b}}}_{2}-{{\rm{a}}}_{2}),\,({{\rm{b}}}_{3}-{{\rm{a}}}_{3})]$$

17 $${\rm{rdERB}}\,(3,\,4)=({{\rm{b}}}_{3}-{{\rm{a}}}_{4})/\,{\rm{\min }}[({{\rm{b}}}_{3}-{{\rm{a}}}_{3}),\,({{\rm{b}}}_{4}-{{\rm{a}}}_{4})]$$

Equations 15–17 employ a normalization by the smaller ERB. This will be favorable if larger overlaps relative to one of the ERBs have to be avoided. Conversely, if large ERB overlaps are desirable, one can use

18 $${\rm{rdERB}}\,(1,\,2)=({{\rm{b}}}_{1}-{{\rm{a}}}_{2})/\,{\rm{\max }}[({{\rm{b}}}_{1}-{{\rm{a}}}_{1}),\,({{\rm{b}}}_{2}-{{\rm{a}}}_{2})]$$

19 $${\rm{rdERB}}\,(2,\,3)=({{\rm{b}}}_{2}-{{\rm{a}}}_{3})/\,{\rm{\max }}[({{\rm{b}}}_{2}-{{\rm{a}}}_{2}),\,({{\rm{b}}}_{3}-{{\rm{a}}}_{3})]$$

20 $${\rm{rdERB}}\,(3,\,4)=({{\rm{b}}}_{3}-{{\rm{a}}}_{4})/\,{\rm{\max }}[({{\rm{b}}}_{3}-{{\rm{a}}}_{3}),\,({{\rm{b}}}_{4}-{{\rm{a}}}_{4})]$$

In principle, one can also use the ERB of the lower (or higher) frequency as reference ERB for normalization. In that case, however, according to Eq. 6 the hearing impairment determines the reference ERB that may render the interpretation of the results more difficult.
